# Metabolic dysfunction-associated steaotic liver disease self-management among the Hispanic/Latino population

**DOI:** 10.3389/fpubh.2025.1499467

**Published:** 2025-04-28

**Authors:** Naomi Hematillake, Mary A. Garza, Emanuel Alcala, Muhammad Y. Sheikh

**Affiliations:** ^1^Department of Public Health, College of Health and Human Services, California State University, Fresno, CA, United States; ^2^College of Health and Human Services, Central Valley Health Policy Institute (CVHPI), California State University, Fresno, CA, United States; ^3^Fresno Clinical Research Center (FCRC), Fresno, CA, United States

**Keywords:** metabolic dysfunction-associated steaotic liver disease, diet, exercise, self-management behaviors, lifestyle-intervention, Hispanic/Latino

## Abstract

**Introduction:**

Hispanic/Latino populations in the U.S. have the highest prevalence of Metabolic Dysfunction-Associated Steaotic Liver Disease (MASLD), and diet and exercise management are crucial for controlling the disease. While quantitative research on general diet and physical activity within this population exists, studies specifically addressing the quantitative analysis of self-management behaviors in Hispanic/Latino individuals with MASLD are notably scarce. This gap underscores the need for our focused investigation into these specific behaviors under the framework of self-management.

**Objectives:**

Our study aims to investigate how various factors such as age, gender, socioeconomic status, and cultural influences are associated with diet and exercise self-management behaviors in Hispanic/Latino individuals with MASLD. We specifically explore the impact of these demographic and cultural factors as independent variables on the dependent variables of diet and exercise self-management behaviors.

**Methods:**

This study involved 94 participants who were recruited from the Fresno Clinical Research Center to participate in a cross-sectional analysis designed to explore diet and exercise self-management behaviors among Hispanic/Latino people with MASLD. Data were collected from January 2023 to February 2023 using a 54-item Qualtrics survey.

**Results:**

The average age of the participants was 53 years. Among the participants, 68.1% self- identified as female, and 80.9% had an annual income of at least $35,000. Age *b* = 0.074, *p* ≤ 0.001, gender *b* = 1.242, *p* ≤ 0.05, and financial stress *b* = 1.887 *p* ≤ 0.01 were predictors for poor exercise self-management behaviors. Disease-related knowledge *b* = −2.264 *p* ≤ 0.001, and familism *b* = −0.344 *p* ≤ 0.05 were predictors for healthy exercise self-management behaviors. There were no significant predictors for diet self-management behaviors among the variables observed in this study.

**Conclusion:**

Age, gender, and financial stress predicted poor exercise self-management behaviors, while disease-related knowledge and familism predicted healthy exercise self-management behaviors.

## 1 Introduction

Chronic liver disease and cirrhosis have emerged as a critical public health concern, ranking as the seventh leading cause of death among Hispanics/Latino people in the U.S. in 2019 (1). Further statistics from 2019 show that about 23% of Hispanic/Latino deaths were liver-related (2). In 2024, studies highlighted that the liver cancer mortality rate among Hispanic/Latino populations was 7.7 per 100,000 [95% CI: 7.5–8.0], which contrasts with rates in white populations at 5.5 per 100,000 [95% CI: 5.4–5.6], in Black populations at 7.6 per 100,000 [95% CI: 7.3–7.8], and in American Indian or Alaskan Native populations at 10.5 per 100,000 [95% CI: 9.1–12.0] (5).Hispanic/Latino people also have the highest prevalence of Metabolic Dysfunction-Associated Steaotic Liver Disease (MASLD), previously known as Non-Alcoholic Fatty Liver Disease (NAFLD) (6), when compared to other ethnic groups in the U.S. (7). The prevalence of MASLD is significantly higher among Hispanic adults in the United States compared to both non-Hispanic populations within the U.S. and populations in other developing countries (8). This indicates a specific health disparity exacerbated by unique socioeconomic and healthcare access issues in the U.S. (55, 56). For instance, Tesfai et al. (8) highlight that Hispanic individuals face a 1.50-fold higher risk of MASLD compared to non-Hispanic individuals. This contrasts with global trends in developing countries where MASLD prevalence is differently impacted by factors such as lower healthcare expenditure and varied cultural practices affecting diet and lifestyle (8). Furthermore, MASLD mismanagement can lead to severe liver diseases like Metabolic Dysfunction-Associated Steatohepatitis (MASH), cirrhosis, and hepatocellular carcinoma (9, 10). These conditions are increasing among Hispanics/Latino people: 25% have MASH, 12% have hepatocellular carcinoma, and 44% have cirrhosis (2). Studies show that diet and exercise management are the best ways to control MASLD and prevent it from worsening (9, 10). Throughout this article diet and exercise behaviors will be referred to as “diet and exercise self-management behaviors.” The use of the term “self-management” denotes the diet and exercise behaviors performed by the patient’s post-diagnosis. Despite the crucial role of diet and exercise in managing MASLD, no quantitative studies have examined diet and exercise self-management predictors among Hispanics/Latino people with MASLD. However available studies on the impacts of diet and exercise behaviors on other metabolic health outcomes, such as diabetes disease management, show improvement (11), Hernandez et al. demonstrated that culturally tailored diabetes self-management programs led to significant improvements in health outcomes, reducing HbA1c levels by an average of 0.5% over 6 months among Hispanic participants. Similarly, Marquez et al. (11) reported that culturally adapted physical activity programs increased weekly moderate to vigorous physical activity by 45 min on average, illustrating the effectiveness of tailored interventions. These findings underscore the potential of customized health interventions to significantly enhance disease management and quality of life for Hispanic/Latino populations diagnosed with MASLD. Regular physical activity not only reduces liver fat but is crucial in managing sarcopenia, often associated with MASLD. Studies have demonstrated the efficacy of exercise in reducing liver fat independently (12) and its critical role in sarcopenia management, emphasizing tailored exercise regimens (13). Additionally, disparities in physical activity levels due to socio-economic factors significantly affect communities, especially in high and middle-income countries, illustrating the broader impacts of inequality on health practices (14). Furthermore, the combination of diet and exercise, along with the use of natural products, has been shown to alleviate sarcopenia, offering a comprehensive approach to managing this condition safely and effectively (15). It is crucial to consider anthropometric measures and laboratory data to monitor sarcopenia, especially given that nearly two-thirds of the study population consists of middle-aged and older adults, who are at a higher risk. These tailored interventions are vital for improving MASLD management and enhancing the quality of life for Hispanic/Latino populations. Previous studies among this population were qualitative and surveyed MASLD awareness and perceptions. Population (7, 16, 17). Limited data available from qualitative studies indicates that various factors, including stress, disease-related knowledge, familism, acculturation, demographic variables (such as age, marital status, gender, race, annual family income, and education level), and disease severity, may influence diet and exercise self-management among Hispanic/Latino individuals with MASLD (16–21, 53, 54). Several types of stress, including health, work, financial, family, and social stress, may impact dietary and exercise self-management among the Hispanic/Latino population, highlighting the importance of evaluating these stressors as they can influence self-management behaviors (22). The low level of MASLD-related knowledge among the Hispanic/Latino population, combined with their increased susceptibility to the disease, underscores the importance of determining the association between MASLD-related knowledge and diet and exercise self-management behaviors (16, 17, 23). Observing the association between acculturation and dietary and exercise self-management among the Hispanic/Latino population is crucial, as studies suggest that acculturation significantly influences these behaviors, potentially impacting MASLD management (3, 17, 20, 24–30). Determining the association between demographic variables (such as age, marital status, gender, race, family income, education level) and disease severity with diet and exercise self-management among the Hispanic/Latino population is vital, as these factors significantly influence these health behaviors; for instance, gender differences show that women tend to exercise less and have more difficulty with dietary management compared to men (31, 32), and individuals with lower socioeconomic status and education levels face increased challenges in self-management (16–21). Identifying the association between immutable demographic variables and self-management behaviors is crucial for determining which populations may require targeted interventions to enhance disease management and prevention strategies. This study aims to fill the gaps in the literature by examining diet and exercise self-management predictors using the “Adherence to Lifestyle Modification Advice in Non-alcoholic Fatty Liver Disease” scale (33). Furthermore, the reliability of this scale, developed by Dubasi et al., has not been tested within this demographic group. Thus, this study also aims to assess the reliability of this scale among Hispanic/Latino populations.

## 2 Methods

### 2.1 Study design

The MASLD Self-Management Among the Hispanic/Latino Population research was approved by the Fresno State Institutional Review Board (IRB) on December 12, 2022. Data were collected from January 2, 2023, to February 13, 2023. The study utilized a 54-item survey administered through a cross-sectional study design to examine the factors influencing diet and exercise self-management among Hispanics/Latinos with MASLD. The variables considered in the study included disease- related knowledge, familism, acculturation, demographic characteristics, disease severity, and stress.

### 2.2 Participant recruitment

A sample size of 94 participants who met the eligibility requirements for the study were recruited out of 206 participants contacted over the telephone and in person at a Gastroenterology and Hepatology research site in Fresno, California. All interviews took place in person or by telephone at FCRC in a secure location that ensured discretion and privacy. The surveys were offered in both Spanish and English. Surveys offered in English were administered to the participants through one-on-one interviews. A Spanish-speaking translator performed surveys offered in Spanish in this interviewer’s presence. Participants were given 10 dollars in cash as an incentive to participate in the study.

### 2.3 Participant inclusion and exclusion criteria

The following inclusion criteria were used for recruited participants: (1) they had to be Hispanic/Latino, (2) diagnosed with MAFLD, and (3) 18 years old or older. Participants were excluded from the study if they were Hepatitis B or C virus carriers.

### 2.4 Measures

All of the questions on the 54-item survey were obtained from previously validated instruments, which were translated into Spanish and administered to participants in both Spanish and English. Questions on demographic characteristics were derived from the California Health Interview Survey (CHIS) and National Health and Nutrition Examination Survey (NHANES) (34, 35). Three items measured demographic characteristics: age, gender, and annual family income. Age was categorized into three groups: 27–46, 47–61, and 62–76. Family income was categorized as ≤ $34,999 and ≥ $35,000. Age and income were treated as continuous variables in bivariate and multivariate analyses. Gender was dummy-coded as “Male” = 1 and “Female” = 0. A modified version of Dubasi and colleagues’.

“Adherence to Lifestyle Modification Advices in Non-alcoholic Fatty Liver Disease” scale was used to examine diet and exercise self-management (33). Self-management was measured using two variables: diet and exercise. Diet included three items, and exercise included two items, each scored on a 5-point Likert scale. Scores ranged from 1 (minimum or never) to 5 (maximum or always) per item. The diet scale was composed of the following three items: ‘How often do you eat fried foods?’, ‘How often do you eta high-salt snacks?’, and ‘How often do you eat refined food items like burgers, pizza, etc.?’. The exercise scale was composed of the following two items: ‘How many days do you exercise in a week?’ and ‘How much time do you exercise for each session?’. This scoring system clarifies that ‘1’ indicates ‘never’ performing the behavior, and ‘5’ indicates ‘always’ performing the behavior, emphasizing proactive self-management of MASLD. The total diet score ranged from 3 to 15, and the total exercise score ranged from 2 to 10. We summed the values of these items to get the total diet score.

Additional questions about substance use, types of exercises, and eating habits were obtained from the nutritional questionnaire administered by the University of Florida Health Center (36) These factors were not included within the analysis due to lack of statistical significance. Questions on disease-related knowledge were modified and derived from a knowledge survey by Ghevariya et al. (37). Disease-related knowledge was measured by the participants’ knowledge of diabetes-causing MASLD. Participants were asked “Which of these conditions do you think can cause fatty liver? (one or more answers) Obesity (1) Diabetes (2) Excess alcohol intake (3) High cholesterol (4)” Responses were dummy-coded as “Yes” = 1 or “No” = 0.

Questions measuring familism were derived from (38) familism scale. Familism was measured by six items covering two components: family support (five items) and subjugation of self for family (three items). Factor analysis grouped these components into one. Items less than 0.400 were eliminated. We did varimax and Promax rotations to confirm our initial factor analysis results, and it confirmed that the loadings did not change. Each item was rated on a 10-point Likert scale from 1 (“strongly disagree”) to 10 (“strongly agree”). Participants were asked “A person should live near his or her parents and spend time with them on a regular basis.” The overall score was the mean of all items, with higher scores indicating higher familism and lower scores indicating lower familism.

Questions measuring acculturation were derived from the 2022 CHIS and the 2019 NHANES. Language use was used as a proxy for acculturation. The question to assess language use was posed as follows “What language(s) do you usually speak at home? Do you speak only Spanish, more Spanish than English, both equally, more English than Spanish, or only English?” Language use had five categories: Only Spanish, More Spanish than English, Both Equally, More English than Spanish, and Only English. Participants who only spoke English were considered more acculturated and responses were dummy-coded as “Only English Speaking” = 1 or “Other” = 0. Questions measuring stress were derived from the Hope Restored Counseling Florida nutritional assessment (39). Financial stress was used to measure stress on a 10-point Likert scale. Financial stress was measured through the following question: “Indicate daily stressors and rate the level of stress from 1 (extremely low) to 10 (extremely high)” compared to other stressors, such as family, work, and social. Higher scores indicated higher stress levels.

Questions on disease severity were derived from the University of Florida Health Center, CHIS, and NHANES (34, 35). Disease severity was measured by the presence of at least one comorbidity in a participant. Comorbidities considered were obesity, diabetes, dyslipidemia, low HDL, sleep apnea, and none. Responses were dummy-coded as “Have at least one comorbidity” = 1 or “None” = 0.

### 2.5 Data analysis plan

All data analysis was performed through the International Business Machines Statistical Package for the Social Sciences (IBM SPSS) Version 29.0.1.1. Power analysis was performed to compute the sample size needed for this study’s bivariate and multivariate analysis tests. In this study, G*Power Factor analysis was employed specifically for determining the required sample size to achieve sufficient statistical power. G*Power Factor analysis provides researchers with the ability to estimate sample size based on the expected effect size, alpha level, and power (40). This is crucial for ensuring that the study is adequately powered to detect statistically significant effects. G*Power does not perform factor analysis; its function is limited to power analysis and sample size calculation. For our factor analysis, which involves exploring the structure of a dataset and calculating Cronbach’s alpha to assess the reliability of the scales, we utilized SPSS. This statistical package allows for detailed factor analysis including the extraction of factors and rotation methods to validate the scales used in the survey. For scale validation, including the computation of Cronbach’s alpha for assessing reliability, we used traditional factor analysis methods conducted through statistical software. These analyses were essential to ensure the internal consistency of the survey scales we adapted and refined for our study. The scale development’s cut-off point was 0.400. A Cronbach’s reliability test was performed to determine if each scale had a minimum Cronbach’s alpha value of 0.6. Simple linear regression analysis, mean, and standard deviation analysis methods were used to examine if a significant relationship existed between the dependent variables, diet and exercise self-management, and the categorical independent variables, disease-related knowledge, acculturation, gender, and disease severity. A Pearson correlation analysis examined the relationship between the dependent variables, diet and exercise self-management, and the continuous independent variables, age, annual family income, familism, and financial stress. The significance of the simple linear regression and Pearson correlation analyses were calculated at the *p*-value ≤ 0.05. Multiple linear regression analyses were used to identify the independent variables significantly associated with diet and exercise self-management. The significance of the multiple linear regression analysis was calculated at the *p*-value ≤ 0.05.

## 3 Results

### 3.1 Sample size and response rate

Power analysis indicated that a sample size of 119 participants was needed; however, we successfully recruited 94 participants from a pool of 208 eligible individuals at the Fresno Clinical Research Center (FCRC). Of these 208 eligible participants, 155 were initially contacted via telephone, resulting in 53 enrollments. Additionally, 56 individuals were approached in person at FCRC, from which 41 agreed to participate. This recruitment process culminated in a total response rate of 45%. The discrepancy in the total number of eligible participants contacted and those who participated involves both those who were unreachable or declined to participate after initial contact.

### 3.2 Demographic characteristics

The participant demographics (*N* = 94) are presented in Table 1. All participants identified as Hispanic/Latino people—most study participants identified as Mexican Hispanic Latino people (74.5%) and female Hispanic Latino people (68.1%). The ages were 27–46 (33%), 47–61 (36.2%), and 62–76 (29.8%), while the mean annual family income was $69,249 (S.D. = $49,198). Notably, nearly 25% of the participants’ families (22.2%) earned less than $35,000 annually (see Table 1).

**Table 1:** Participant demographics, *N* = 94.

Demographic	Group	N	%	Mean	SD	Min	Max
Gender	Male	30	31.9%				
	Female	64	68.1%				
Age	27 to 46	31	33.0%				
	47 to 61	32	36.2%				
	62 to 76	28	29.8%	53	12.6	27	76
Family income	≤ 34,999	18	22.2%				
	≥ 35,000	63	77.8%	$69,249	$49,198	$6,000	$200,000

^ (Missing 1 Value) *N* = 93. ^^ *N* = 81.

### 3.3 Factor and reliability analysis

Factor analysis was performed to refine three scales: (a) diet self-management, (b) exercise self-management, and (c) familism (see Table 2). The original diet self-management behaviors scale was 15 items and the exercise self-management behaviors scale contained 10 items. Following factor analysis, the diet scale comprised three items. The diet scale had a Cronbach alpha value of 0.640. Possible scores for diet behaviors ranged from a minimum of three to a maximum of 15. The exercise scale comprised two items, with a Cronbach alpha value of 0.789. Possible scores for exercise behaviors ranged from two to 10. Higher scores on both the diet and exercise scales indicated poorer health behaviors. Familism was measured using six items, which assessed two components: family support and subjugation of self for family. Initially, five items measured family support and three items measured subjugation of self for family. However, after factor analysis, these two components were combined into one component, with each of the six items measuring family support and subjugation of self for family on the same scale.

**Table 2:** Factor analysis for diet, exercise, and familism scales.

Factor	Items	Cronbach alpha
Diet Scale		
Self-management behaviors	How often do you eat fried foods? How often do you eat high-salt snacks? How often do you eat refined food items like burgers, pizza, etc.?	0.640
Exercise Scale		
Self-management behaviors	How many days do you exercise in a week? How much time do you exercise for each session?	0.789
Familism Scale		
Family Support and Subjugation of Self for family	A person should often do activities with his or her immediate and extended families, for example, eat meals, play games, go somewhere together, or work on things together A person should live near his or her parents and spend time with them on a regular basis or share a house A person should always support members of the extended family, for example, aunts, uncles, and in-laws, if they are in need, even if it is a big sacrifice A person should always defend his or her family’s honor no matter what the cost Aging parents should live with their relatives A person should help his or her older adult parents in times of need, for example, help financially	0.776

### 3.4 Summary of findings

The simple linear analysis showed associations between exercise self-management and several variables: age (*b* = 0.074, 95% CI [0.029, 0.118], *p* ≤ 0.001), gender (*b* = 1.242, 95% CI [0.034, 2.449], *p* ≤ 0.05), and financial stress (*b* = 1.887, 95% CI [0.602, 3.171], *p* ≤ 0.01), all statistically significant. Conversely, disease-related knowledge (*b* = −2.264, 95% CI [−3.481, −1.047], *p* ≤ 0.001) and familism (*b* = −0.344, 95% CI [−0.672, −0.015], *p* ≤ 0.05) were significantly associated with better exercise self-management outcomes. No statistically significant predictors were found for diet self-management among the variables observed in this study (see Table 3).

**Table 3:** Multiple linear regression for exercise behaviors.

95% confidence interval			
Variables	B	Lower bound	Upper bound
Constant		0.093	−0.627
Demographic			
Age	0.074***	0.029	0.118
gender	1.242*	0.034	2.449
Income	0.602	−0.818	2.023
Disease severity			
Have at least one comorbidity	1.444	−0.249	3.137
Disease-Related Knowledge			
Knowledge that diabetes causes NAFLD	−2.264***	−3.481	−1.047
Stress			
Financial stress	1.887**	0.602	3.171
Familism			
Family support and subjugation of self for family	−0.344*	−0.672	−0.015
Acculturation			
Only English Speaking	−0.031	−1.254	1.193

*p*-value * ≤ 05, ** ≤ 01, *** ≤ 001.

## 4 Discussion

The primary findings of this study suggested that older age and high financial stress were predictors of poor exercise self-management. Males had worse exercise self-management than females. High disease-related knowledge and scores for family support and subjugation of self on the familism scale predicted healthy exercise self-management. None of the observed variables in this study (age, gender, annual family income, disease-related knowledge, disease severity, stress, familism, acculturation) predicted diet self-management among Hispanic/Latino people with MASLD.

### 4.1 Diet self-management

Although none of the variables examined in this study were significant predictors for diet self-management among the Hispanic/Latino population with MASLD, literature that has examined diet self-management indicated that variables such as disease-related knowledge, acculturation, and familism may be significant to diet self-management (16–21). The lack of significance observed in this study may be due to the scale not being tested among the Hispanic/Latino population before, thus leaving the possibility of decreased cultural appropriateness. No scales are available to measure MASLD diet and exercise management, specifically among Hispanics/Latinos. Therefore, this study utilized the Dubasi and colleagues’ questionnaire to assess adherence to diet and exercise behaviors for weight management due to its validity as a scale among the general population (33). Further research should examine how to culturally tailor the diet scale and determine what variables may influence diet self-management among Hispanics/Latinos with MASLD.

### 4.2 Financial stress on exercise self-management

This study demonstrated that increased financial stress predicts poor exercise self-management behaviors among Hispanics/Latinos with MASLD. This finding is significant as it identifies a previously unrecognized factor influencing MASLD management. Existing research on the impact of financial stress on exercise among overweight Hispanic/Latino individuals indicates that 53% of those sampled did not prioritize exercise (41). Recent studies have suggested that Hispanics/Latinos have contended with increased financial burdens because of the COVID-19 pandemic (22). Therefore, financial stress may predict poor exercise self-management because of the sudden financial burden experienced among Hispanics/Latinos following the COVID-19 pandemic (22). Other research has indicated that financial stress may predict poor exercise self-management because people with MASLD tend to have multiple comorbidities associated with increased financial hardship, such as obesity, diabetes, etc. (42). These findings underscore the importance of targeting financial stress as a predictor for MASLD and metabolic health management among Hispanics/Latinos. Qualitative analysis can further explore perceptions and beliefs about the effects of financial stress on exercise self-management in this population, providing insights for intervention development. Interventions addressing financial stress should include stress management programs and assess their impact on exercise self-management among Hispanic/Latino individuals with MASLD.

### 4.3 Gender, age, disease-related knowledge, and familism on exercise self-management

This study found that Hispanic/Latino males with MASLD had poorer exercise self-management compared to females (*p* ≤ 0.05). This contrasts with research on the general Hispanic/Latino population, which shows men have healthier exercise self-management often due to more physically demanding occupations (28, 29, 43). Further research should explore if men with MASLD work in less labor-intensive jobs, leading to poorer exercise self-management, or if additional factors impact their exercise self-management. This study found that older age predicted poor exercise behaviors among Hispanic/Latino people with MASLD (*p* ≤ 0.001). No other studies have examined this predictor among this specific group. However, findings align with research on the general Hispanic/Latino population, which also links older age with poor exercise self-management (44). Different age groups within the Hispanic/Latino population may have varying reasons for exercise behaviors, suggesting that interventions should be tailored to age-specific needs (44, 45). High knowledge of diabetes as a cause of MASLD predicted healthier exercise self-management among Hispanics/Latinos with MASLD (*p* ≤ 0.001). This is consistent with qualitative studies suggesting increased disease-related knowledge improves health outcomes (16, 17, 23). Similar trends are observed in literature on obesity, diabetes, and metabolic disorders (46, 47). Promoting education on MASLD causes and benefits of exercise should be included in future interventions (4, 17, 48). High scores on the familism scale significantly predicted healthy exercise self-management among Hispanics/Latinos with MASLD (*p* ≤ 0.05). This finding aligns with qualitative studies that suggest increased familial support motivates healthier exercise behaviors (16, 17, 23). Including family support in MASLD interventions could improve exercise behaviors in this population. This study found that high scores for subjugation of self for family predicted healthy exercise self-management among Hispanic/Latino people with MASLD (*p* ≤ 0.05). No literature examined this predictor among this group, so findings were compared to studies on Hispanic/Latino people with obesity. Previous research suggested that high subjugation of self for family scores predicted poor exercise self-management and weight struggles among those with obesity (31, 32, 49). This study’s findings differ, possibly because subjugation of self and family support were measured together. Future research should investigate these components separately. Additional studies offer promising avenues for more effective MASLD management among Hispanic/Latino populations through the exploration of biomarkers such as Sirtuin 1 and dietary interventions, however this specific focus extends beyond the current project’s scope, setting a direction for future research initiatives (50–52). Furthermore, beyond the traditional pillars of exercise and diet, which are foundational in managing sarcopenia by maintaining muscle mass and function, there is growing evidence supporting the integration of natural products (15). These products offer unique bioactive compounds that not only complement the muscle-maintaining benefits of diet and exercise but also provide innovative mechanisms to combat the aging-related decline in muscle quality (15). According to Tarantino et al. (15), such products are gaining recognition for their safety and availability, suggesting a promising area for further research and application in lifestyle interventions.

## 5 Conclusion

The research presented highlights that older age and financial stress contribute to poor exercise self-management in Hispanic/Latino individuals with MASLD, noting particularly worse outcomes among males compared to females. This study’s insights into the roles of disease-related knowledge, familial support, and socio-economic factors enrich the discussion on targeted lifestyle interventions. These findings prompt future studies to refine interventions that cater specifically to the unique demographic and cultural aspects of the Hispanic/Latino community, ensuring the effective management of MASLD. Further investigations could explore additional variables that influence diet self-management, an area where this study found no significant predictors.

### 5.1 Limitations

This study had three main limitations. First, participants were self-selected to participate in the study, impacting randomization. Second, response bias was possible but controlled by the interviewer staying on topic and asking survey questions as written. Third, recall bias was possible, as some questions relied on participants recalling their self-management behaviors. This factor was controlled by asking participants to recall behaviors within the same week or day of the interview. Additionally, despite conducting a power analysis that suggested a sample size of 119 was required to achieve sufficient statistical power, the study ultimately recruited 94 participants. This shortfall in reaching the desired sample size may have impacted our ability to detect statistically significant differences, particularly for diet self-management among Hispanic/Latino individuals with MASLD. While the lack of significance in some findings might be attributed to the cultural appropriateness of the scales used, it is also plausible that the specific items retained for assessing physical activity and dietary behaviors were not robust enough to capture the nuances of these behaviors effectively. Future studies should consider both refining the items on these scales and ensuring a larger sample size to enhance the power to detect meaningful differences.

### 5.2 Strengths

This study’s primary strength is its originality, as no other quantitative research has examined predictors of diet and exercise behaviors among Hispanic/Latino people with MASLD. Additionally, it successfully recruited a difficult-to-reach population with a high prevalence of MASLD and increased liver mortality risk. Including Spanish-translated materials and services during data collection, they ensured cultural sensitivity, clear communication, and accuracy. Lastly, the scales used to examine diet and exercise self-management had not been tested among Hispanics/Latinos before, so this study also served to test their reliability in this population.

## Data Availability

The raw data supporting the conclusions of this article will be made available by the authors, without undue reservation.
